# Diseases Peculiar to Females

**Published:** 1871-04

**Authors:** J. P. Logan

**Affiliations:** Atlanta, Ga.


ATLANTA

### MEDICAL & SURGICAL JOURNAL.

NEW SERIES.

Vol. IXB. APRIL, 1871. No. 1.

### DISEASES PECULIAR TO FEMALES.

BY J. P. LOGAN, M. D., Atlanta, Ga.

At your special request I have concluded to contribute an occasional article upon Uterine Pathology and Therapeutics, for your Journal.

In consenting thus to appear before the profession, I do not wish to be regarded as considering myself an authority upon the diseases of females, but rather as a seeker after knowledge in this direction, and enter upon their discussion as much for my own benefit, in acquiring accurate and definite views as from any hope of enlightening others.

It is true that I have been giving some special attention to the pathology and therapeutics of these diseases for some years, and believe that I have learned something beyond the mere routine of former days, and yet I can but feel that we have even now attained to very little comparatively that can be regarded as fixed and definite. Finding such a wide field for discussion and so much that is admitted to be undecided, I hope it will not be regarded as presumptuous in so humble a worker as myself, to endeavor to aid in some small degree, in advancing uterine therapeutics, if in no other way, by presenting from time to time the status of the most eminent gynæcologists of this country and Europe, in regard to some of the most important questions which force themselves continually upon the general practitioner, whether he will or no; compelled as he is for obvious reasons, to assume the responsibility of treating the mass of diseases of this class, arising within the range of his own regular professional labours. It is a melancholy and I might say alarming fact that a very large proportion of our females, in what are called the better classes of society in the southern part of the United States at least do not go through child-bearing even a single time, without becoming the subject of some one of the many forms of uterine trouble, and in a great many instances disease of this character is developed in all classes, and in those who have never been subjected to this trying ordeal, and in not a few who have barely attained the age of maturity.

In this state of affairs, no medical man who has any sense of his obligations to humanity, or of the responsibilities of his profession, can be indifferent to this subject, however much he may be discouraged or disgusted with the result of his efforts to come to some correct understanding of the true causes, the essential pathology and treatment of these maladies.

Judging others by myself, then, any co-laborer in this most difficult field of professional uncertainty will be welcomed, and even the small effort which I shall make in this direction may not be found unacceptable to some of your readers as possibly tending to elicit some facts in regard to the many vexed and unsettled questions in this range of investigation.

As a justification for this intimation in regard to the unsatisfactory results of treatment, and the entirely unsettled views of even the most enlightened members of our profession (and those who are justly regarded as authorities upon this subject) upon some of the most important points in the modern treatment of female diseases, I will refer to the absolutely conflicting views of such men as Sims, Emmet, Nott, Thomas, Barker, Peaslee, and others of our most eminent investigators in this department, as exhibted in a late discussion before the Medical Society of the county of New York upon the subject of Intra-uterine Medication.

*The occasion of the discussion was the presentation of a paper by Dr. J. C. Nott, in which reference was made to the leading points of an article published by himself in the Obstetric Journal for 1869, upon the various indications for intra-uterine treatment, the various means devised to meet them,

*Medical Record and Half Yearly Abstract of the Medical Sciences.

and especially a new and safe instrument for uterine injection—in connection with which he stated, among numerous other things, that most of the agents commonly introduced into the uterus in the topical treatment of its diseases have a greater or less coagulating effect upon blood and other matters usually found in its cavity, the important points being that some kind of chemical reaction takes place between these agents and the fluids found in the uterus, and that this must greatly modify the effect of their application, and that such coagulated masses were sometimes retained in the uterine cavity for an indefinite time, producing great irritation and protracted discharges. He thought that the favorite method of making these applications in New York, by means of a probe wrapped with cotton and dipped into a solution of the medicament, or Dr. Sims' plan of using a curved glass rod without the cotton, to which not more than two or three drops could adhere, must be in most cases either negative or hurtful, the greater part of the substance upon the probe being rubbed off in passing the cervical canal, the remainder being rendered inert by the albuminous fluid found in the womb, or by coming into contact with sound membrane from flexion of the uterus, produce injury; or, by meeting with some point of exposed vessels, produce the most violent uterine colic, or even metritis or ovaritis. He favored the injection of suitable substances into the uterus for disease of its cavity, first cleaning it out by suction with a syringe; or, if the discharge be too tenacious to be removed in this way, by employing a weak solution of muriatic acid or common salt, or some other solvent of albuminous matter. He gave the result of various agents he had used in a series of experiments upon this subject, and condemned, in the most decided terms, the use of the nitrate of silver and the chromic and carbolic acids, but recommended in strong terms iodine, as not interfering with the fluidity of the albuminous matter, as better tolerated by the uterus than any other potent remedy, and as being something more than a stimulant, a caustic, or a styptic—being a remedy *sui generis*, but especially valuable on account of its ready absorption, in chronic inflammation, where the object was to affect the deeper tissues of the uterus. In conclusion, he stated that gynæcologists might be divided into three schools—the cutting school, the cauterizing school, and those who use no intra-uterine treatment, but depend upon vaginal injections, hygiene and constitutional remedies—and that while he had often heard it charged that Drs. Sims and Emmet, who were generally regarded as the representatives of the cutting school, too frequently used the knife and scissors, he had rarely seen bad results from their operations, and believed, from actual observation, that caustics often do more harm than comes from cutting the cervix.

Dr. Fordyce Barker regarded it as especially fitting that a full discussion of the subject should take place in a city where intra-uterine medication might almost be said to have originated, and where it had been pushed to a more audacious extent than anywhere else. He favored the use of sulphate of zinc made into a paste with glycerine, and introduced through a canula into the cavity of the uterus, and had used it for fifteen years, and had found no unpleasant symptoms resulting. This he had found to be especially useful in the trying cases of menorrhagia so frequently associated with the climacteric period.

Dr. Peaslee gave it as his opinion that intra-uterine medication should be resorted to in comparatively very few cases, and that the character of these cases, as well as the substances to be used, are entirely unsettled, and that the practice at present was merely empirical. If the plan is adopted at all, he preferred such substances as iodine, tannin, sat. solution, per sulphate of iron, and alum, to so powerful a substance as chromic acid; while Dr. Emmet has used the latter for fifteen years, and is its decided advocate.

Dr. Kammerer did not hesitate to use intra-uterine injections when necessary, but usually confined his medication to the canal of the cervix, and always injects tepid water into the cavity of the body as a means of cleansing it, even when he only intends treating the canal of the cervix. He regards the simple dilatation of the internal orifice, repeated once or twice a week, as very efficient in the treatment of disease of the cavity, gradually diminishing the hyper-secretion in catarrh of the body in some, even without any local application, but always resorts to intra-uterine remedies when the discharges are muco-purulent.

Dr. Whitehead advocated the introduction of strong tincture of iodine, (Dr. Budd's formula, 80 grs. of iodine, half a drachm of iodide of potassium and one ounce of alcohol), upon a cotton wrapped probe to the fundus, in endo-metritis and Dr. Jacobi had often employed undiluted carbolic acid, without any ill effects, but does not regard nitrate of silver as efficient on account of its too purely local action.

Dr. Byrne had after a long experience with the injection of caustics into the cavity of the uterius, abandoned their use and now employed such mild substances as sulphate of soda, tannin &c., which easily return from the uterus if a proper catheter be employed. He did not however rely upon local treatment in cases of troublesme intra uterine affection, being satisfied that there is always a constitutional difficulty, and that no topical treatment is of permanent advantage, without reference to this pathological view and the most careful attention to constitutional and hygienic measures. Dr. Gaillard Thomas, (admitted in Europe, as well as in this country, to be high authority upon any subject connected with female diseases), gave it as his impression that intra-uterine injections constituted no advance in the treatment of uterine disease, that they have done and are doing a great deal of harm, and although popular, their evil results will cause them after a more thorough trial to be discarded.

He narrated some cases in which *fatal* results had attended this practice, though admitted that the cases were rare and could not alone decide the propriety of the operation, but contended that the risk was too great to justify the employment of any such questionable means for the relief of an annoying affection, from which the danger of death is, as a rule, very remote. He advocated the dilatation of the cervix, thus offering free egress to any morbid discharges, the introduction of the medicated cotton wrapped probe if necessary, and the internal administration of ergot and tonics.

In conclusion I would state very briefly that my own experience is not favorable to the intra-uterine injections of any character whatsoever, so little indeed as to have determined me, for the present at least, to abandon their use for the more safe plan, suggested by Dr. Thomas. The preparation which I now introduce upon the cotton wrapped probe is composed of equal parts, (which can be varied by a larger proportion of iodine as the organ becomes accustomed to the application), of tinc. of iodine, pure glycerine and fluid extracts of opium and belladonna. My plan is also to deplete the enlarged, and engorged uterus, (which pathological conditions you find in almost all cases of endometritis), by the free application of pure glycerine upon dentists cotton carried up to the os uteri, in such bulk as can be introduced through the speculum, with the view of securing its retention for twenty four hours,from which I obtain a copious discharge of serum and thus materially aid in reducing the bulk and weight of the organ as well as in removing the irritability which invariably attends upon this morbid condition.

It will be recollected that the glycerine treatment is not original with myself, but was first suggested by Dr. J. Marion Sims, the distinguished pioneer in uterine surgery. I also, in the intervals between the applications, direct the daily free irrigation of the os uteri and vagina, with *hot* water of as high temperature as can be *gradually* borne, which as I have reason to believe materially aids in restoring the parts to a state of health, and certainly renders the patient much more comfortable.

But as Dr. Byrne suggests in the discussion referred to, no simply local treatment can meet the demands of a large majority of the cases which come under the charge of the medical man.

But as it was not my object in this paper to do more than introduce the general subject of female diseases, I will forbear, intending probably to present in some more distinct form the varied results of my own experience, through the columns of your journal at some future time.

